# Supplementary material for: Alterations in Growth Habit to Channel End-of-Season Perennial Reserves towards Increased Yield and Reduced Regrowth after Defoliation in Upland Cotton (Gossypium hirsutum L.)
Source: Int J Mol Sci. 2023 Sep 16;24(18):14174. doi: 10.3390/ijms241814174 (PMC10532291; doi:10.3390/ijms241814174)
Supplement: Supplementary file 1 [file ijms-24-14174-s001.zip › Supplemental Figures.pdf]

Figure S1. Plots showing population distribution of expression traits recorded for *FT* (A), *FUL* (B), *LFY* (C), *AP1* (D), and *SOC1* (E) at developmental stage S1 in 2019.

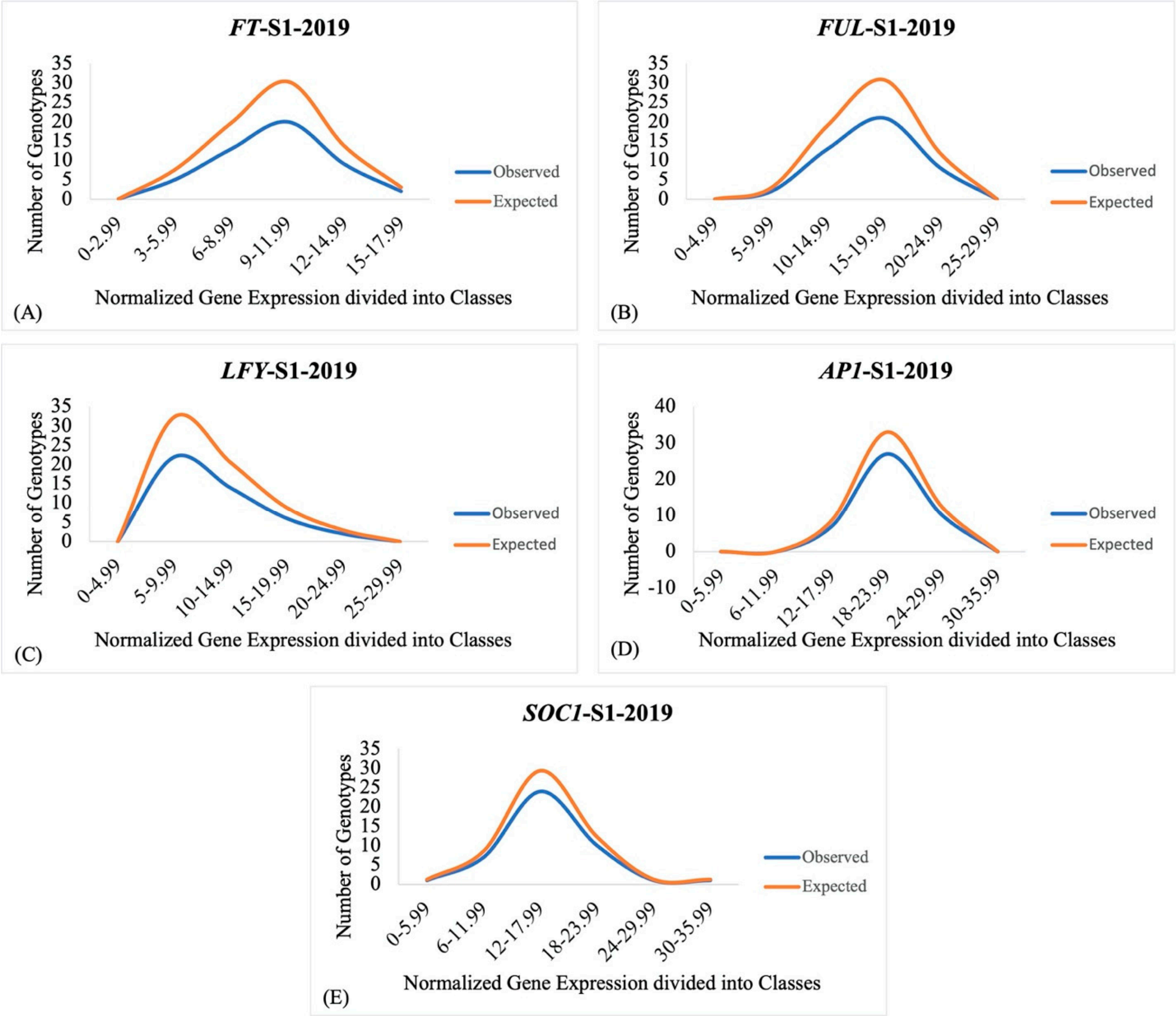

Figure S2. Genomic distribution of the expression trait-associated SNP markers on the cotton chromosomes. An expected number of SNPs per chromosome was calculated and plotted with the observed number of SNPs per chromosome to show the biased distribution of SNP markers on different cotton chromosomes.

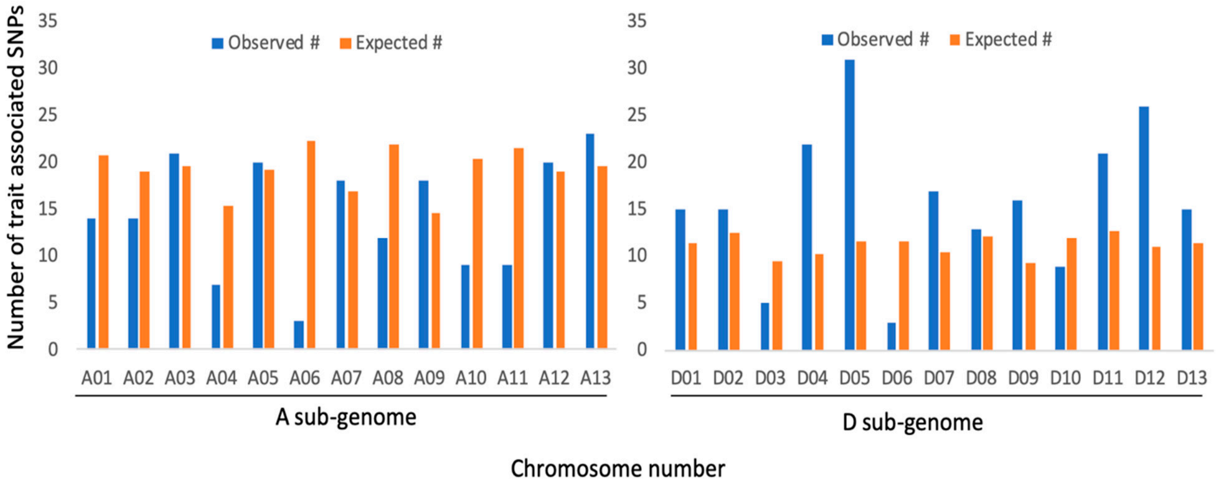

Figure S3. Picture showing Linkage Disequilibrium (LD) blocks of associated markers on the cotton A and D subgenomes.

<u>A subgenome</u>

A01

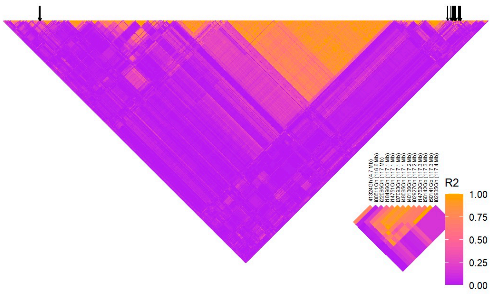

A02

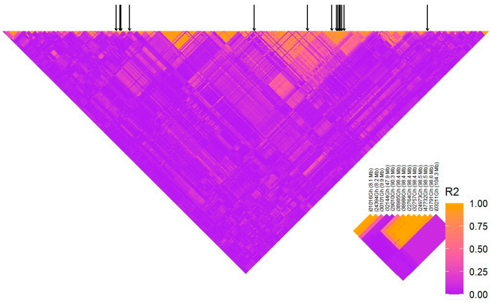

A03

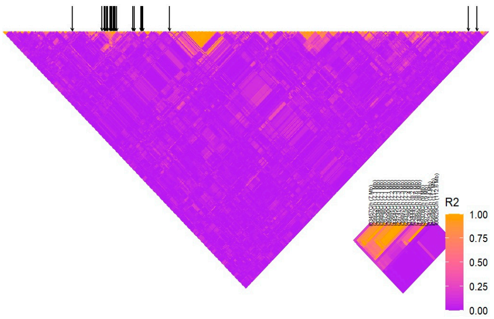

A04

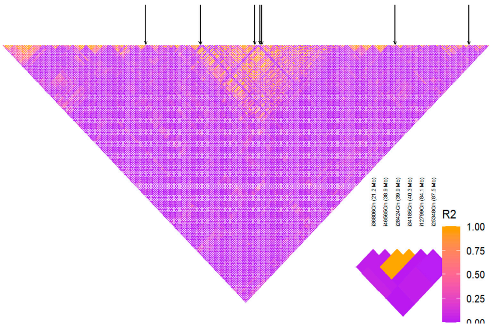

A05

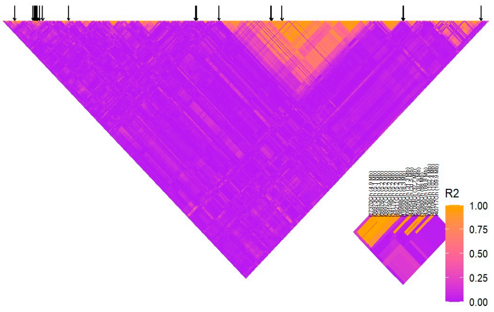

A06

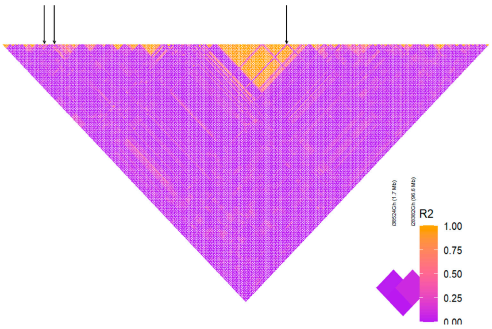

A07

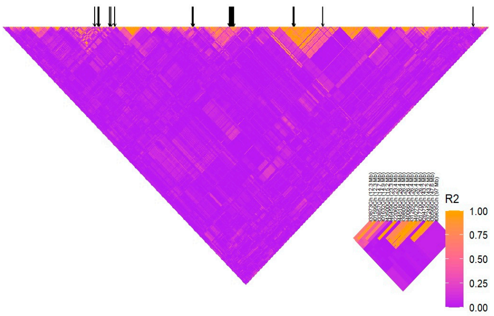

A08

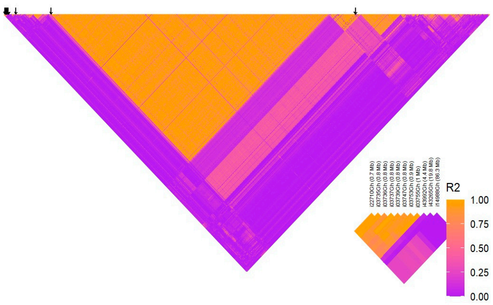

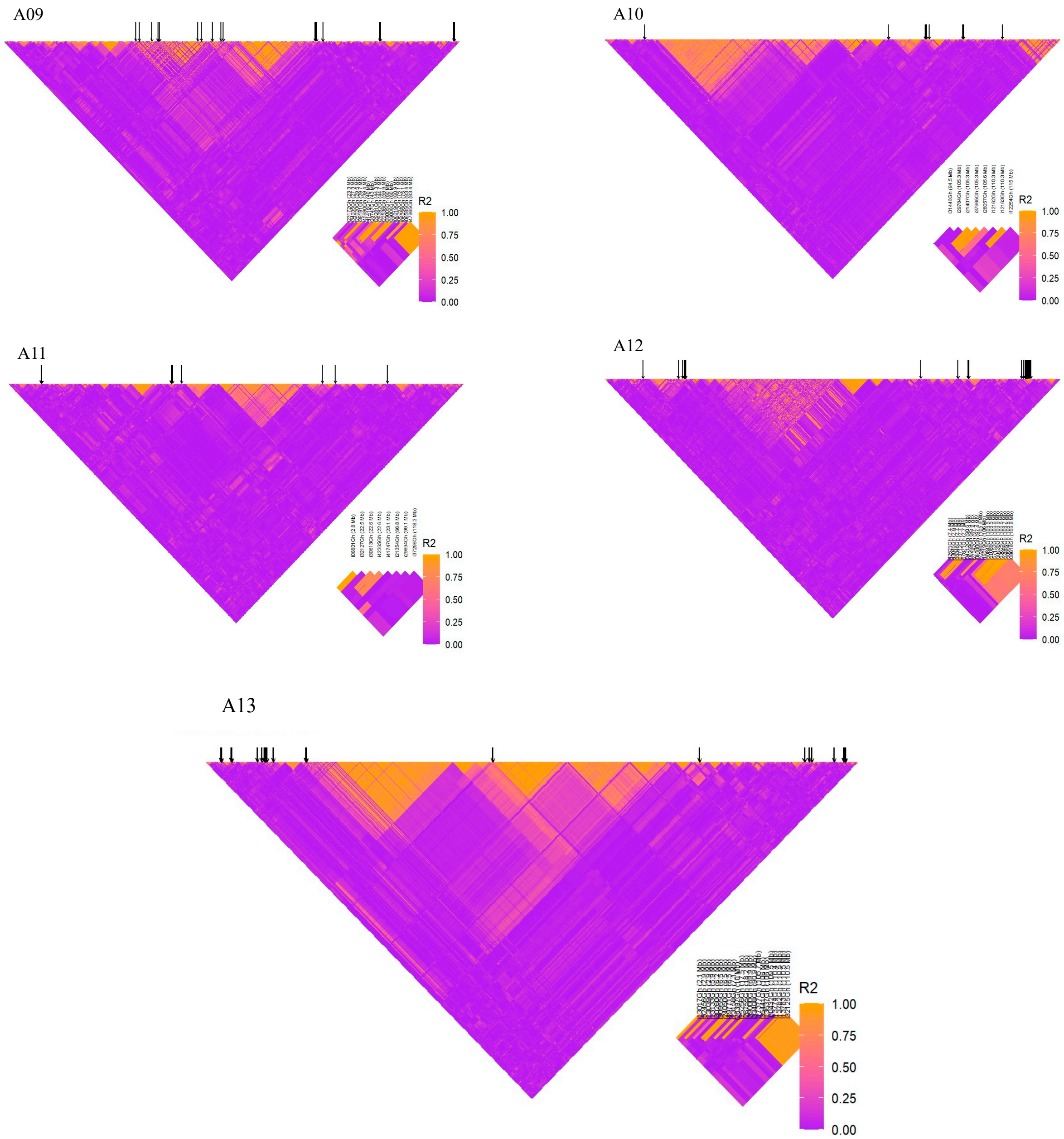
A09
A10
A11
A12
A13
R2
1.00
0.75
0.50
0.25
0.00

D subgenome

D01

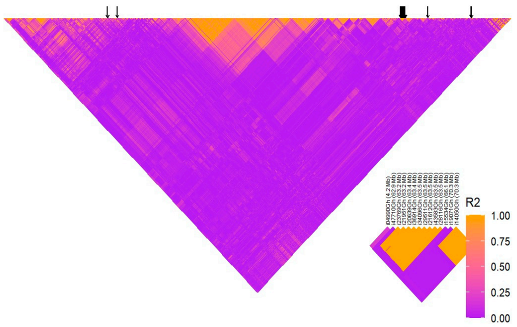

D02

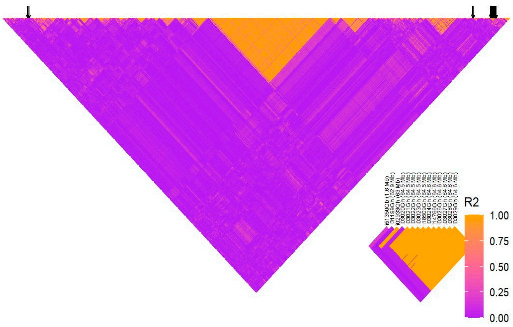

D03

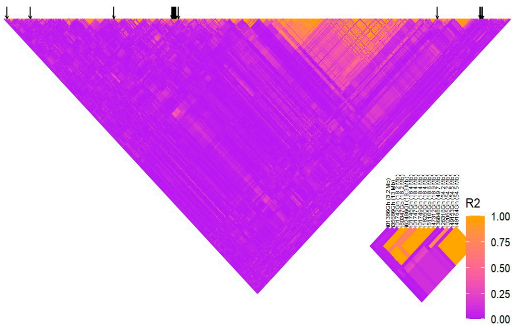

D04

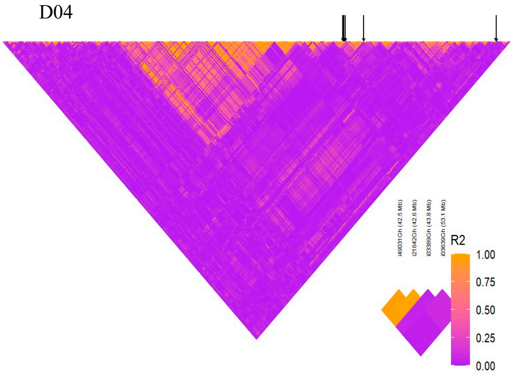

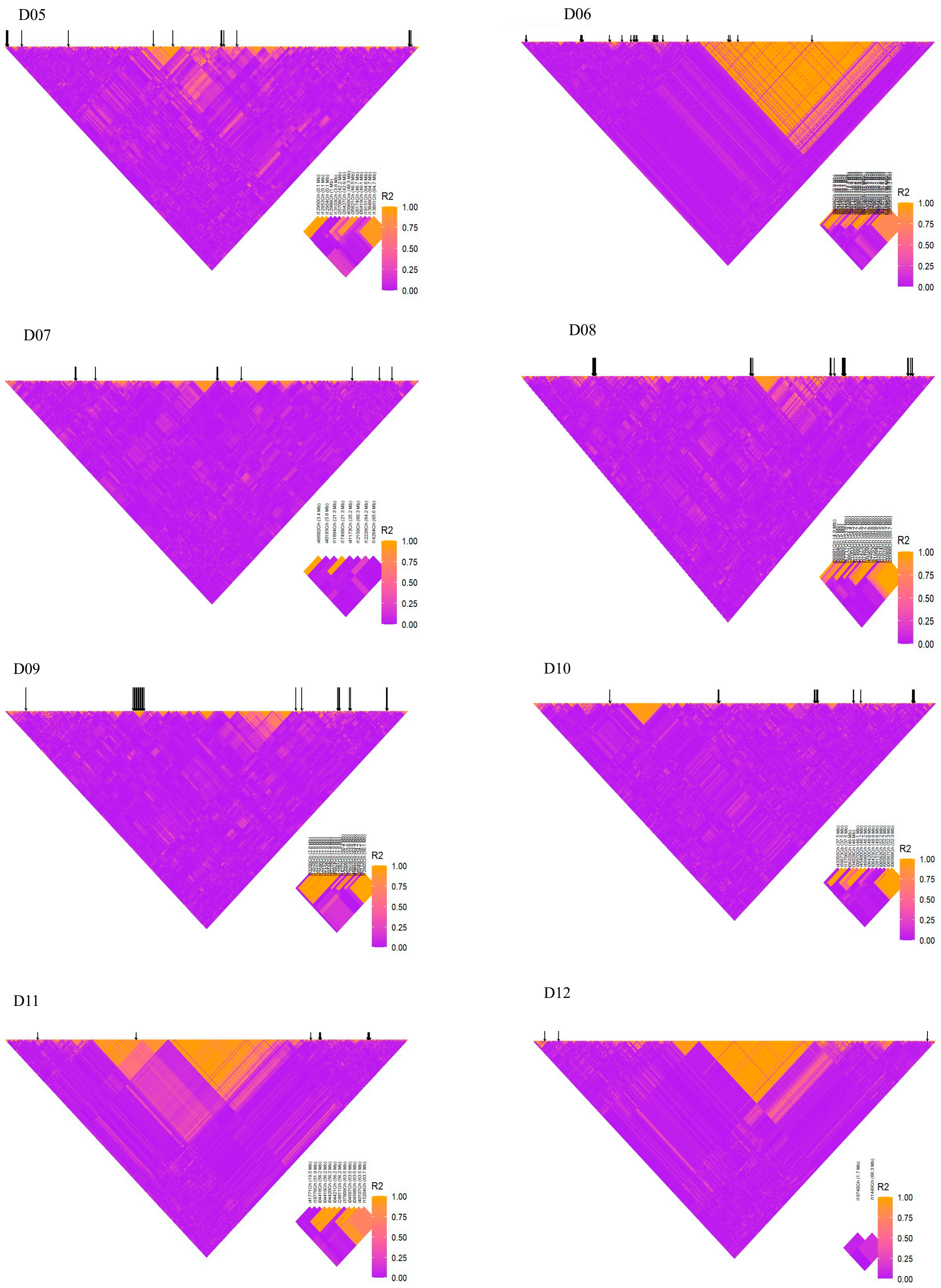
D05
D06
D07
D08
D09
D10
D11
D12
R2
1.00
0.75
0.50
0.25
0.00

D13

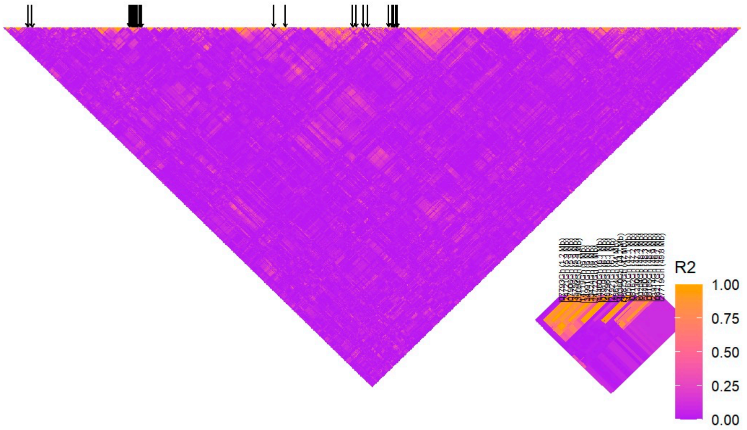

Figure S4. Principal Component Analysis of 44 Upland cotton genotypes of the mini-core collection. Most of the genotypes belong to Population 1, and the accessions which are close to each other were perceived as similar [1] on both PC1 and PC2. The genotypes in the positive ordination may be utilized for a heterosis breeding program. Almost zero clustering was noted in the genotypes representing Population 3, and the negative or near to zero ordination suggests their very low fitness for the heterosis breeding program.

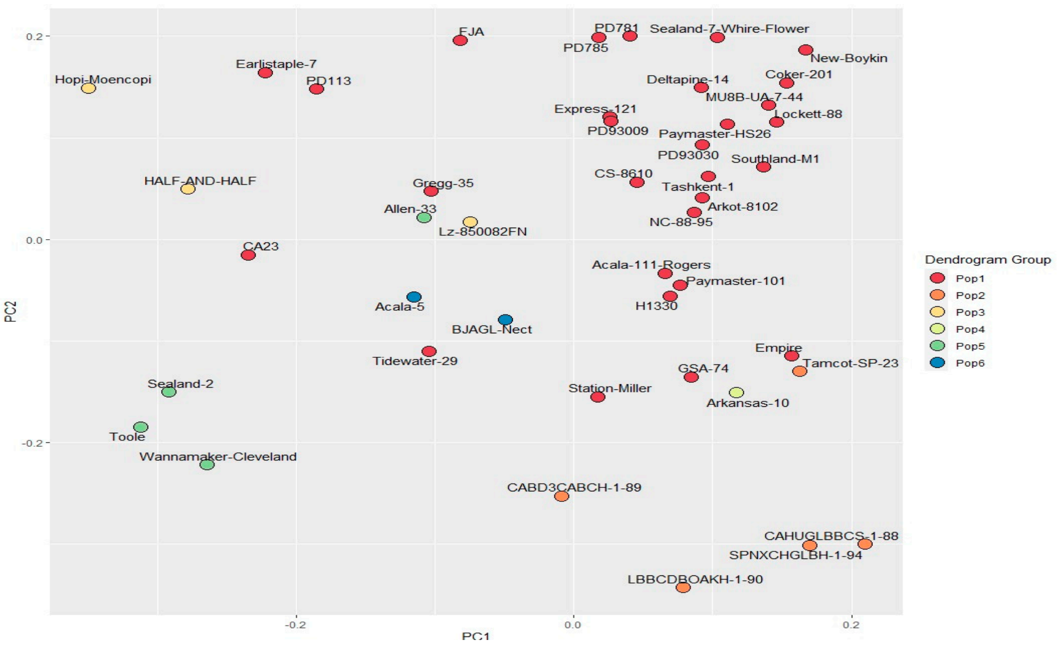

Figure S5. Pedigree diagram of 44 Upland cotton mini-core collection lines. Yellow nodes represent the mini-core collection genotypes, whereas grey nodes represent the parental genotypes. Note: Lines are drawn to show the complex relationship of these genotypes.

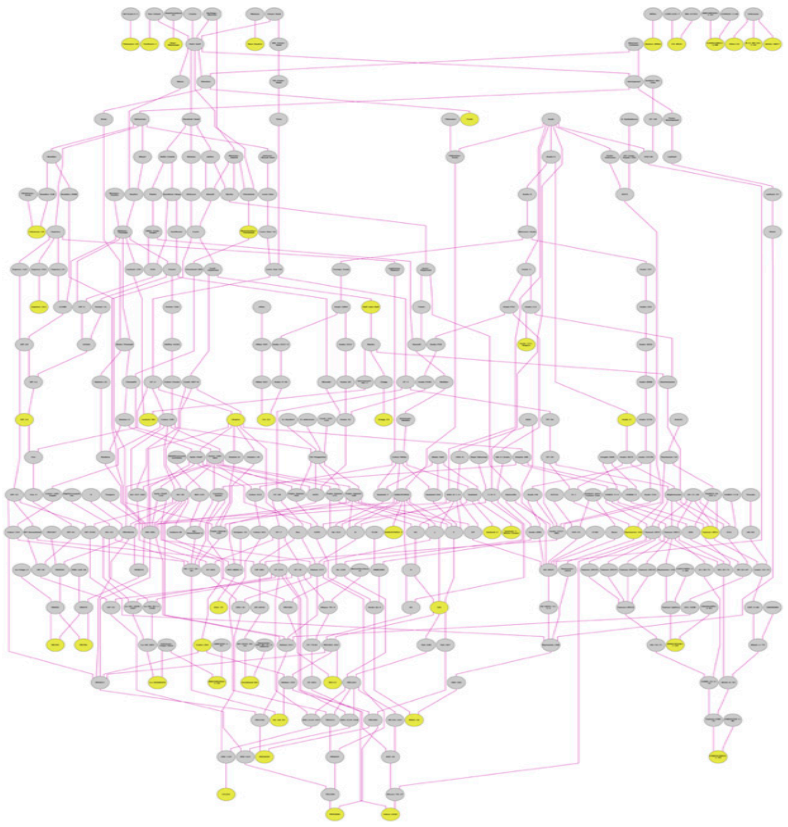

Figure S6. Phylogenetic tree of *SOC1*, *AP1*, and *FUL* genes developed using Clustal omega, A. The evolutionary distance is depicted next to each branch. Details of the gene family members of *FT* and *LFY* are shown in B.

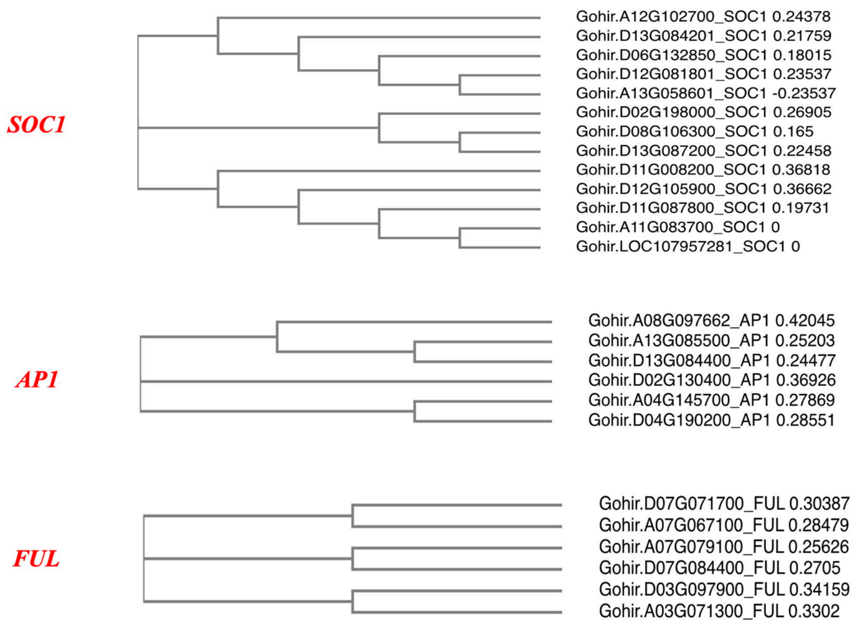

(A)

Gene ID	Gene Name	Chromosome	Location
Gohir.A08G227700	*FT*	A08	125104463 to 125101112
Gohir.D08LOC107909115	*FT*	D08	67103565 to 67100103
Gohir.D07G050800	*LFY*	D07	6018761 to 6014246
Gohir.A07G046500	*LFY*	A07	5562304 to 5557891

(B)

Figure S7. Common transcription factor binding sites (TFBS) for the GATA-TF family in the promoter regions (1Kb) of five floral induction and meristem identity genes (*FT*, *SOC1*, *LFY*, *FUL*, and *AP1*).

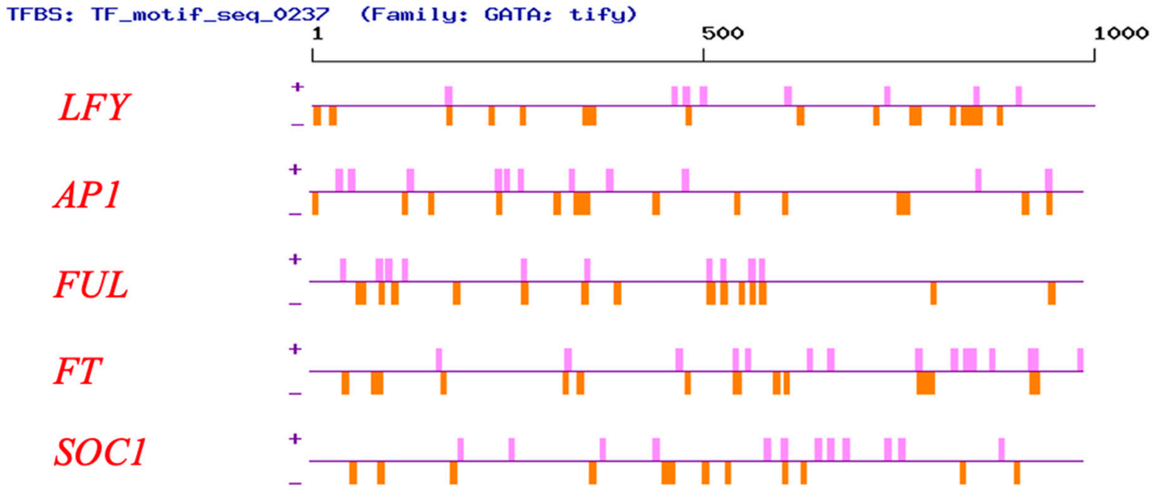

Figure S8. Determination of the molecular functions of the *trans*-eQTLs and the putative causes of epistatic interactions between the eQTLs and the genes of interest in the Upland cotton genome (A and D subgenomes). Note: Rounded rectangle shows common miRNA matches among gene bodies of the *FT, LFY, AP1, SOC1,* and *FUL* gene family members and the eQTLs.

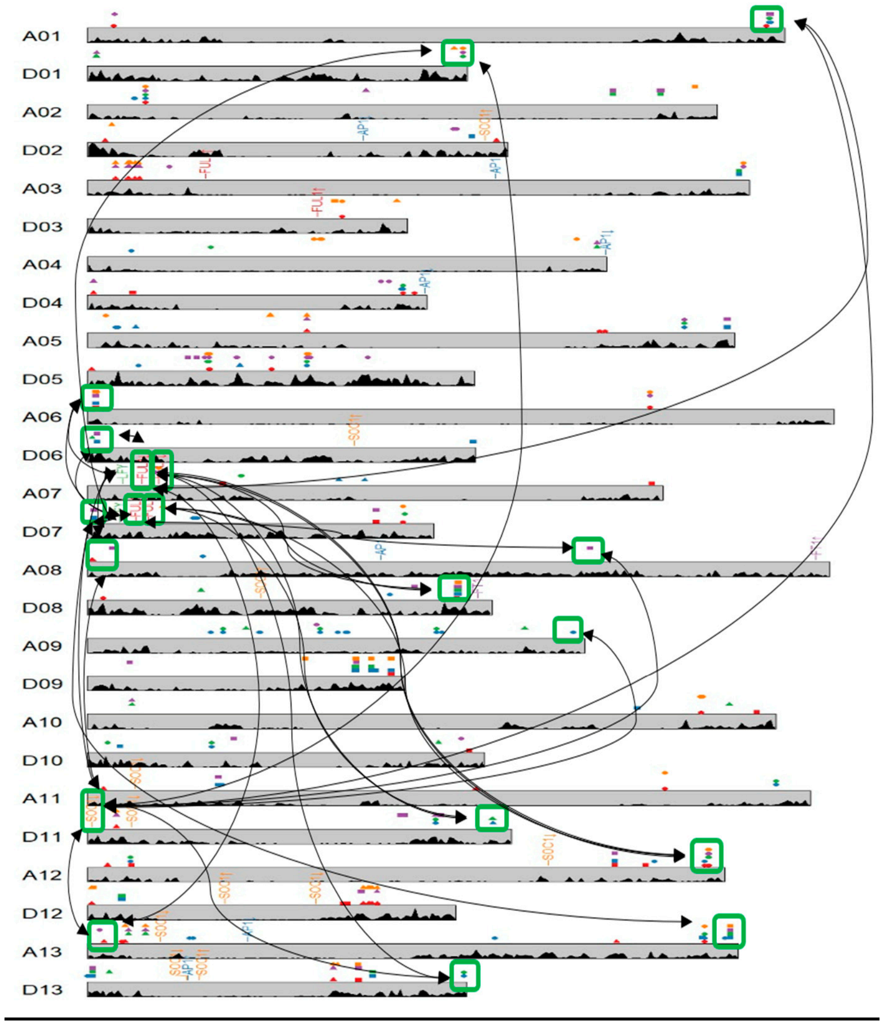

Figure S9. CpG islands in the gene bodies of *Gohir.D07G050800* (A) and *Gohir.A07G046500* (B).

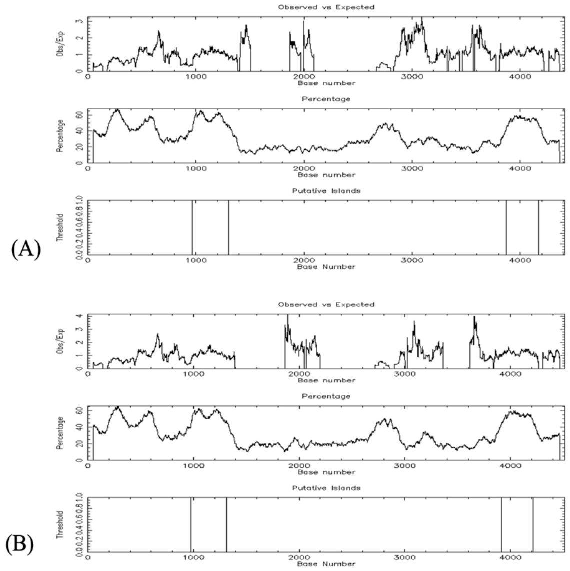

References

1. Rathinavel, K. Principal Component Analysis with Quantitative Traits in Extant Cotton Varieties (*Gossypium hirsutum* L.) and Parental Lines for Diversity. *Curr. Agric. Res. J.* **2018**, *6*, 54–64. https://doi.org/10.12944/carj.6.1.07.
