# Supplementary material for: Alterations in Growth Habit to Channel End-of-Season Perennial Reserves towards Increased Yield and Reduced Regrowth after Defoliation in Upland Cotton (Gossypium hirsutum L.)
Source: Int J Mol Sci. 2023 Sep 16;24(18):14174. doi: 10.3390/ijms241814174 (PMC10532291; doi:10.3390/ijms241814174)
Supplement: Supplementary file 1 [file ijms-24-14174-s001.zip › Supplementary Tables.pdf]

Table S6. List of the 44 Upland cotton mini-core collection genotypes cultivated for three years from 2017 to 2019.

Genotype ID	Genotype name
1	ACALA 111 ROGERS
2	ACALA 5
3	ALLEN 33
4	ARKANSAS 10
5	ARKOT 8102
6	BJAGL NECT
7	CA23
8	CABD3CABCH-1-89
9	CAHUGLBBCS-1-88
10	COKER 201
11	CS-8610
12	EARLISTAPLE 7
13	EMPIRE
14	EXPRESS 121
15	FJA
16	GREGG 35
17	GSA 74
18	H1330
19	HALF AND HALF
20	HOPI MOENCOPI
21	LZ.850082FN
22	LBBCDBOAKH-1-90
23	LOCKETT 88
24	M.U.8B UA 7-44
25	NC 88-95
26	NEW BOYKIN
27	PAYMASTER 101
28	PAYMASTER HS26
29	PD 0113
30	PD 781
31	PD 785
32	PD 93009
33	PD 93030
34	SEALAND #2
35	SEALAND #7 WHITE FLOWER
36	SOUTHLAND M1
37	SPNXCHGLBH-1-94

38	STATION MILLER
39	TAMCOT SP-23
40	TASHKENT 1
41	TIDEWATER 29
42	TOOLE
43	WANNAMAKER CLEVELAND
46	DELTAPINE 14

Table S7. List of primers used in this study.

Primer	Strand	Sequence
FT	F	CTACACCTTGGTTATGGTGGATC
	R	CACGAACACAAAACGATGGA
SOC1	F	AGCATGCAGTGGCAGCATCTGA
	R	TGGCTCTGACGCGGGTTACG
FUL	F	AGGAAATGACCCATCAGCCAC
	R	GCTGCACTAGGATTACCCTCTTC
LFY	F	GCAGTGTCGGGATTTCTTGATT
	R	AGGCAATGTAGGGCGTAGCAAT
AP1	F	GTGATGCTGAGGTCGCTTTGAT
	R	ATGGACCAGTTGCCCTGAGATT
ACT4-2	F	GGAATGGTGAAGGCTGGTT
	R	TATCATCCCAGTTGCTGACG
